# Supplementary material for: Exploring the influence of social media use motivations on acculturation orientations and psychological adaptation among Chinese students in Malaysia
Source: PLoS One. 2025 May 9;20(5):e0323407. doi: 10.1371/journal.pone.0323407 (PMC12064037; doi:10.1371/journal.pone.0323407)
Supplement: Attachment — Submitted filename: Abstract.docx [file pone.0323407.s001.docx]

**Abstract**

-The abstract mentions that motivations (information seeking, social, and entertainment) improve psychological adaptation but does not specify which motivations are most influential. Providing this detail could enhance understanding.

-You need to mention the software you used for the study

-The phrase "help Chinese students improve their psychological adaptation in Malaysia" is repeated in slightly different forms. Condensing these repetitions can improve readability.

The upcoming content is very good.
