# Supplementary material for: Exploring the influence of social media use motivations on acculturation orientations and psychological adaptation among Chinese students in Malaysia
Source: PLoS One. 2025 May 9;20(5):e0323407. doi: 10.1371/journal.pone.0323407 (PMC12064037; doi:10.1371/journal.pone.0323407)
Supplement: Attachment — Submitted filename: Rebuttal Letter.docx [file pone.0323407.s003.docx]

**Rebuttal Letter**

Dear Editors,

In response to PLOS ONE's decision regarding the manuscript titled "Exploring the Influence of Social Media Use Motivations on Acculturation Orientations and Psychological Adaptation Among Chinese Students in Malaysia" (Decision: Revision Required [PONE-D-24-52159]. I would like to express my sincere gratitude to the editor and reviewers for their invaluable and constructive comments. These insights have been instrumental in refining and enhancing this new version of the submitted paper.

All the comments were considered providing the following changes to the points raised by the academic editor and reviewers (all of them are highlighted in the marked-up copy):

*“Journal Requirements:*

*1. Please ensure that your manuscript meets PLOS ONE's style requirements, including those for file naming.”*

**Action(s) performed:** Changes have been made according to the submission guidelines and PLOS ONE's style requirements, including those for file naming and the reference formatting.

2. *Please include a complete copy of PLOS’ questionnaire on inclusivity in global research in your revised manuscript.*

**Action(s) performed:** Yes, questionnaire on inclusivity in global research had been done.

3. *Please provide additional details regarding participant consent.*

**Action(s) performed:** I sincerely appreciate your valuable suggestions. Yes, additional details regarding participant consent had been done, refer to p.11.

4. *“We noticed you have some minor occurrence of overlapping text with the following previous publication(s), which needs to be addressed”*

**Action(s) performed:** Thank you for drawing my attention to the issue of overlapping texts. I understand the importance of being original and avoiding plagiarism in academic publications.

To clarify, the manuscript you are referring to is my previously published paper that focuses on the relationship between social media use and cultural orientations. The goal of the paper submitted to PLOS is to incorporate psychological adaptation as a dependent variable into the model and cultural orientation as a mediating variable to expand and deepen the understanding of the phenomena studied.

The overlapping texts mainly appear in the introduction and methodology. To solve this problem, I plan to revise the manuscript to reduce any overlapping texts. Specifically, I have carried out the following tasks:

I. Rewrite overlapping paragraphs to highlight unique aspects of the new research.

II. Reasonable citations to ensure academic norms.

III. Make sure the results and discussion sections focus on new findings and insights.

If there are any additional concerns or further clarification needed, please do not hesitate to let me know. I am committed to ensuring the manuscript meets the highest standards of academic integrity.

5. *Please state what role the funders took in the study.*

**Action(s) performed:** ] Thank you for pointing this important things. The funders had no role, and some statement had been added, refer to p.22.

6. *Authors must share the “minimal data set” for their* submission*.*

**Action(s) performed:** Thank you for pointing this out. The minimal data set had been uploaded to figshare. (DOI: 10.6084/m9.figshare.28379111)

7. *Please review your reference list to ensure that it is complete and correct.*

**Action(s) performed:** I sincerely appreciate your comments. The reference list had been checked carefully. I am sure that the list is complete and correct.

**Review Comments**

*- “Reviewer 1: 1. The abstract mentions that motivations (information seeking, social, and entertainment) improve psychological adaptation but does not specify which motivations are most influential. Providing this detail could enhance understanding.*

**Action(s) performed:** I fully agree with your perspective. The relevant information has been added in the abstract. [The results demonstrated that three types of use motivations can help Chinese students improve their psychological adaptation in Malaysia, while entertainment motivations have the greatest influence on psychological adaptation. refer to p.1].

*- “Reviewer 1: 2. “You need to mention the software you used for the study.”*

**Action(s) performed**: Thank you for pointing out this important things. The information about software was added in the abstract.[A total of 438 questionnaires were analyzed by using SPSS and AMOS. refer to p.1].

*- “Reviewer 1: 3. The phrase "help Chinese students improve their psychological adaptation in Malaysia" is repeated in slightly different forms. Condensing these repetitions can improve readability.*

**Action(s) performed:** Thank you for pointing this out. To enhance the fluidity and readability, I have undertaken a thorough revision of the paper's language.

*- “Reviewer 2: 1. The study focuses exclusively on Chinese students in Malaysia, limiting the generalizability of findings to other international student groups or host countries. This limitation should be more explicitly acknowledged in the conclusion.*

**Action(s) performed:** I fully agree with the comments. The limitation has been explicitly acknowledged in the conclusion. [First, the study only focused on the Malaysian context, which makes its findings difficult to apply to Chinese students studying in other Asian countries. Again, as the study only included Chinese students, it is not representative of international students from other countries in Malaysia. To achieve more generalized results, it is crucial to examine more diverse and representative samples. Refer p. 20)

*- “Reviewer 2: 2. The literature review is comprehensive but leans heavily on studies conducted in Western contexts. Including more references from Asian or Malaysian studies would improve regional relevance.*

**Action(s) performed:** Thank you for pointing this out. To address your concern and enhance the regional relevance of the literature review, I have taken the initiative to incorporate additional references from Asian and Malaysian studies. The specific references are as follows:

A study on Chinese students in Singapore suggested that online information, including daily life information and academic resources, facilitated their adaptation [47]. Refer to p.5

Similarly, Guo et al. (2014) found that Chinese international students in Japan utilized social media for social-informational purposes, which enhanced their life satisfaction and contributed to a sense of happiness [48]. Refer to p.5

Chinese students’ attitudes towards Islamic culture positively influenced their psychological adaptation in Malaysia [67]. Refer to p.7

Rich resources on social media present Chinese culture from multiple perspectives, which can help Southeast Asian students better learn Chinese language, understand cultural differences, and reduce cultural conflicts [83]. Refer to p.8.

*- “Reviewer 2: 3.* Strengthen the discussion of the rejected hypotheses (H7a, H7b, H10a, H10b) by providing theoretical or empirical justifications.

**Action(s) performed:** I fully agree with your perspective. Further elaboration were added in the discussion. [Entertainment usage had no significant effect on two types of cultural orientations, potentially attributed to several factors. Firstly, Asian students prefer American, Korean, and Japanese entertainment content [88]. Thus, it is plausible that entertainment motivations do not significantly influence Malaysian and Chinese cultural orientations. Secondly, social media platforms often tend to prioritize popular or trending content under the influence of algorithmic recommendations [116,117], which may not always adequately reflect cultural diversity and uniqueness. This distribution mechanism may lead to a narrow range of entertainment content that users are exposed to on social media, thus limiting their understanding and experience of different cultures. Furthermore, digital media has accelerated the exchange and integration of global cultures, fostering a trend of cultural homogenization worldwide [118]. Cultural homogeneity may lead to the disappearance of local cultural characteristics [119]. Refer to p.19].

H8, H9, and H10 demonstrate that acculturation orientations can explain how social media use motivations affect psychological adaptation. This also suggests that the effect of social media use motivations on psychological adaptation is not direct but happens through some intermediate mechanism. Specifically, in the influence of information and social motivations on psychological adaptation, both Malaysian and Chinese cultural orientations play a mediating role. However, the two cultural orientations did not mediate the relationship between entertainment motivations and psychological adaptation. Therefore, the extent to which Chinese international students use social media for entertainment motivations is directly associated with their psychological adaptation in Malaysia without a significant indirect effect through Malaysian and Chinese cultural orientations. This is attributable to the fact that entertainment usage had no effect on cultural orientations. Therefore, cultural orientations cannot serve as an effective mediator to explain the relationship between entertainment motivations and psychological adaptation. Refer to p.19]]

*- “Reviewer 2: 4. The manuscript notes that respondents use both Chinese (e.g., WeChat) and Malaysian (e.g., WhatsApp) platforms but does not explore how platform-specific usage influences adaptation. This could add depth to the analysis.*

**Action(s) performed:** Thank you for pointing out. My study did not explore how platform-specific usage influences adaptation, which could add depth to the analysis. Due to time and resource constraints, this aspect was not fully addressed in the current research. However, your review comments have not only helped me identify the shortcomings in my paper but also pointed out the direction for improvement, for which I am deeply grateful.

*- “Reviewer 2: 5. Highlight the potential implications of specific social media platforms (e.g., cultural proximity in WeChat vs. global networking in WhatsApp).*

**Action(s) performed:** Thank you very much for your valuable comments on my research. The potential role of specific social media platforms (such as WeChat and WhatsApp) in influencing cultural orientation and psychological adaptation, as you mentioned, is indeed a topic worthy of further exploration. In the study, I primarily focused on the impact of social media use motivations on cultural orientation and how this, in turn, affects the psychological adaptation of Chinese international students in Malaysia. While I did not directly compare the effects of different social media platforms, your suggestion provides me with a new perspective. I will seriously consider your suggestions and apply them in my future research.

*- “Reviewer 2: 6. Consider revising the conclusion to emphasize the broader applicability of findings while acknowledging limitations more explicitly.*

**Action(s) performed:** Thank you very much for the valuable suggests. The limitation has been explicitly acknowledged in the conclusion. [First, the study only focused on the Malaysian context, which makes its findings difficult to apply to Chinese students studying in other Asian countries. Again, as the study only included Chinese students, it is not representative of international students from other countries in Malaysia. To achieve more generalized results, it is crucial to examine more diverse and representative samples. Refer p. 20]

*- “Reviewer 2: 7. The visual presentation of findings, such as path analysis and conceptual frameworks, could be more detailed and visually engaging.*

**Action(s) performed:** Thank you for pointing this out. The graphs and tables in the article have been embellished to make them more visually appealing and provide more information.

For example, the figure of conceptual framework has been modified to ensure that all key variables, paths, and hypotheses are clearly marked. In addition, explanatory notes are added to help readers understand the information presented in the figures.

*- “Reviewer 2: 8. There are occasional grammatical errors and awkward phrasing.*

**Action(s) performed:** Thank you for pointing this out. The full text has been further modified to ensure that there are no grammatical errors and awkward phrasing.

*- “Reviewer 3: 1. 1)The abstract should better emphasize the urgency and significance of the research, particularly its relevance to the growing population of international students. 2) Include details on the sampling method, and 3)briefly highlight the implications of the findings. Avoid vague language and strengthen the concluding statements to emphasize the study’s practical applications.*

**Action(s) performed:** Thank you very much for the valuable suggests. 1) Some information about the urgency and significance of the research has been added in the abstract. [As the world becomes increasingly globalized and communication and transportation systems rapidly develop, the number of international students is growing remarkably. Consequently, the influence of social media on the psychological adaptation of international students has become a matter of concern. However, the relationship between social media and psychological adaptation remains inconclusive. Moreover, there is a scarcity of studies investigating the influence of social media use motivations on the psychological adaptation of international students. Refer to p.1]

2) Details on the sampling method has been added. [An online survey was administered, employing the convenience sampling technique to collect data from a sample of Chinese international students in Malaysia. Refer to p.1]
3) Briefly highlight the implications of the findings and strengthen the concluding statements to emphasize the study’s practical applications. Relevant information has been added in the abstract. [The findings suggested that Chinese students can use social media purposefully to obtain a better psychological adaptation in Malaysia. Besides, social media designers can enhance the functions and services of social media to provide more support for international students. Relevant departments and educators in Malaysia should appreciate the role of social media and acculturation orientations in helping international students have a better overseas experience and enhance Malaysia’s competitiveness in the international education market. Refer to p.1]

*-“Reviewer 3: 2. Language and Grammar:The manuscript contains grammatical and syntactical errors that hinder readability.*

**Action(s) performed:** I sincerely appreciate your valuable suggestions. The full text has been further modified to ensure that there are no grammatical errors and awkward phrasing.

*- “Reviewer 3:3. Methodology: 1)Sampling Method. The manuscript does not clearly specify the sampling technique; 2)The justification of distributing the questionnaire online; 3) Clearly explain the rationale for adapting the scales and the specific modifications made to ensure validity in the current context.*

**Action(s) performed:** Thanks for your thorough review and valuable suggestions.

1) Specific information on sampling methods is described in the methodology section. [Due to Chinese students' broad distribution across Malaysian institutions and large geographical span, generating a comprehensive sampling frame is difficult. Additionally, the specific number of Chinese students at each university is unknown to the researcher. Thus, probability sampling is unsuitable for this study. Instead, the study adopted the convenience sampling method. refer to p.11]

2) Content about the advantages of using online questionnaires have been added. [With the proliferation of the internet and social media, online surveys have become an efficient and convenient method of data collection.Compared with the traditional method (e.g., paper-pencil), online questionnaire distribution is a very time-saving method. It can reach a broader range of samples, thereby facilitating the collection of a larger sample size [94]. refer to p.11-12]
3) I have added a table in the revised manuscript that lists each dimension of the scales and the corresponding items.

*- “Reviewer 3: 4. Findings and Preliminary Analysis. Include descriptive statistics (e.g., skewness, kurtosis) to confirm the normality of the data, as CB-SEM requires normally distributed data.*

**Action(s) performed:** Thanks for your valuable suggestions. I added the descriptive statistics for skewness and kurtosis to verify the normality of our data. [When performing Structural Equation Modeling (SEM) analysis, it is essential to examine the skewness and kurtosis of the variables to verify compliance with the normal distribution assumption [106]. If the absolute values of skewness and kurtosis fall within the range of 0 to 1, the data can be considered approximately normally distributed [107]. In the current study, the absolute values of skewness and kurtosis are below 1 (see Table 2), indicating that the data in the current study were normally distributed. Refer to p.14]

In addition, I have added multicollinearity diagnosis to ensure that there are no collinearity problems between the variables. [Additionally, evaluating multicollinearity is essential before using SEM to analyze data. The common method to identify multicollinearity issues is to check the variance inflation factor (VIF) and tolerance (TOL) values. A variable with a VIF value greater than ten and a TOL value less than 0.10 indicates that the variable is highly correlated with other variables [106]. As the data shown in Table 1, all the VIF value was less than 10, and the TOL value was higher than 0.1. Thus, multicollinearity was not an issue for this study. Refer to p.14]

*- “Reviewer 3: 5. 1)Expand on the theoretical implications by linking findings more explicitly to acculturation theory and cross-cultural adaptation literature; 2)Practical implications should offer actionable insights for stakeholders such as educators, social media designers, and policymakers; 3)The discussion should explicitly address the rejected hypotheses.*

**Action(s) performed:** I sincerely appreciate your valuable suggestions. 1) Some information has been added on the theoretical implications by linking findings more explicitly to cross-cultural adaptation literature. [First, this research makes substantial contributions to the field of cross-cultural adaptation. It stands as one of the pioneering studies to explore the relationship between social media use motivations, acculturation orientations, and the psychological adaptation of Chinese international students within Asian countries. By emphasizing the significance of considering mediators when assessing the influence of social media usage on psychological adaptation, this study facilitates a deeper understanding of the underlying mechanisms that influence the cross-cultural adaptation process of international students. Furthermore, by drawing the attention on the Asian context—an area that has received relatively scant attention compared to Western contexts—this research enriches the geographical diversity of cross-cultural adaptation literature. It establishes a more comprehensive and diversified landscape in the domain of cross-cultural adaptation studies. Refer to p.20]

2) Actionable insights have been offer. [To be specific, designers should develop features that streamline the process of finding relevant and accurate information for international students. This could involve integrating advanced search functionalities, creating dedicated content hubs for international students, or partnering with educational institutions and local organizations to provide curated content. In view of the fact that entertainment use has a significant effect on psychological adaptation, but has no effect on cultural orientation. Thus, social media designers can incorporate diverse and culturally relevant content, such as cultural variety shows, short videos, interactive games, and quizzes, which can promote cultural understanding and learning. Refer to p.21]

Malaysian policymakers and educators should consider using popular social platforms such as WeChat and WhatsApp to connect with Chinese students to learn about their needs and offer support. Considering the positive impact of Malaysian cultural orientations on psychological adaptation,, relevant departments can set up a special section for international students on social media platforms. This section would serve as a hub for disseminating knowledge about Malaysian culture and values, facilitating exchanges with international students and increasing intercultural understanding. Refer to p.21]

3) Further elaboration were added in the discussion. [Entertainment usage had no significant effect on two types of cultural orientations, potentially attributed to several factors. Firstly, Asian students prefer American, Korean, and Japanese entertainment content [88]. Thus, it is plausible that entertainment motivations do not significantly influence Malaysian and Chinese cultural orientations. Secondly, social media platforms often tend to prioritize popular or trending content under the influence of algorithmic recommendations [116,117], which may not always adequately reflect cultural diversity and uniqueness. This distribution mechanism may lead to a narrow range of entertainment content that users are exposed to on social media, thus limiting their understanding and experience of different cultures. Furthermore, digital media has accelerated the exchange and integration of global cultures, fostering a trend of cultural homogenization worldwide [118]. Cultural homogeneity may lead to the disappearance of local cultural characteristics [119]. H8, H9, and H10 demonstrate that acculturation orientations can explain how social media use motivations affect psychological adaptation. This also suggests that the effect of social media use motivations on psychological adaptation is not direct but happens through some intermediate mechanism. Specifically, in the influence of information and social motivations on psychological adaptation, both Malaysian and Chinese cultural orientations play a mediating role. However, the two cultural orientations did not mediate the relationship between entertainment motivations and psychological adaptation. Therefore, the extent to which Chinese international students use social media for entertainment motivations is directly associated with their psychological adaptation in Malaysia without a significant indirect effect through Malaysian and Chinese cultural orientations. This is attributable to the fact that entertainment usage had no effect on cultural orientations. Therefore, cultural orientations cannot serve as an effective mediator to explain the relationship between entertainment motivations and psychological adaptation. Refer to p.19]

*- “Reviewer 3: 6. Reformat all references according to the journal’s guidelines.*

**Action(s) performed:** Changes have been made according to the submission guidelines and PLOS ONE's style requirements, including reference formatting.

*-“Reviewer 3:7. Data Availability:To comply with PLOS ONE policies, ensure that raw data is publicly available or accessible upon request.*

**Action(s) performed:** Thank you for pointing this out. The minimal data set had been uploaded to figshare. (DOI: 10.6084/m9.figshare.28379111)

Sincerely yours

*Wenwen Zhao*
